# Social Medicine: Twitter in Healthcare

**DOI:** 10.3390/jcm7060121

**Published:** 2018-05-28

**Authors:** Yash Pershad, Patrick T. Hangge, Hassan Albadawi, Rahmi Oklu

**Affiliations:** 1 Department of Vascular and Interventional Radiology, Minimally Invasive Therapeutics Laboratory, Mayo Clinic, Phoenix, AZ 85054, USA; hangge.patrick@mayo.edu (P.T.H.); albadawi.hassan@mayo.edu (H.A.); oklu.rahmi@mayo.edu (R.O.); 2 Department of General Surgery, Mayo Clinic, Phoenix, AZ 85054, USA

**Keywords:** social media, Twitter, communication, patient–physician relationships, technology, public health

## Abstract

Social media enables the public sharing of information. With the recent emphasis on transparency and the open sharing of information between doctors and patients, the intersection of social media and healthcare is of particular interest. Twitter is currently the most popular form of social media used for healthcare communication; here, we examine the use of Twitter in medicine and specifically explore in what capacity using Twitter to share information on treatments and research has the potential to improve care. The sharing of information on Twitter can create a communicative and collaborative atmosphere for patients, physicians, and researchers and even improve quality of care. However, risks involved with using Twitter for healthcare discourse include high rates of misinformation, difficulties in verifying the credibility of sources, overwhelmingly high volumes of information available on Twitter, concerns about professionalism, and the opportunity cost of using physician time. Ultimately, the use of Twitter in healthcare can allow patients, healthcare professionals, and researchers to be more informed, but specific guidelines for appropriate use are necessary.

## 1. Introduction

Social media enables the public sharing of information between anyone with an account. While previously used primarily to connect public figures such as politicians, celebrities, and athletes with the general public, additional uses of social media have arisen. Calls to improve the alienating and inaccessible language of healthcare in order to improve patient–physician communication are not unique, and continue to plague medicine [1]. Thus, improving the transparency and accessibility of information in medicine is critical. Since social media allows for the sharing of information, applications of social media to medicine have recently garnered a great deal of attention [2]. Currently, Twitter is the most popular form of social media used for healthcare communication [3].

Conflicting opinions have stirred controversy about if and how medical professionals should use social media platforms in their work. Skeptics of social media in healthcare cite the potential for misinformation, conflicting advice, and unprofessionalism as evidence that social media is not an appropriate medium on which to share healthcare information [4]. Some argue that social media has no place in healthcare, while others claim that the open sharing of information enabled by social media would revolutionize accessibility to medicine. Here, we examine the use of Twitter in medicine and pose the question: “In what capacity should Twitter be used for the sharing of information in healthcare?”

To answer this question and analyze uses of social media in medicine, we first examine how medical professionals have used digital media in the past. Then, we compare Twitter’s features to prior media to investigate how Twitter is currently being used in medicine. From its current uses, we derive its limitations and potential for growth; finally, these limitations guide proposed solutions about how to effectively leverage Twitter in medicine. Ultimately, a review of the relevant literature demonstrates that the use of Twitter to share information about treatments and research among physicians has the potential to greatly improve care. However, when used inappropriately (e.g., for direct public interactions between patients and doctors), Twitter’s privacy and misinformation concerns outweigh the benefit of accessibility.

## 2. The History of Digital Media and Healthcare

Previous uses of digital media provide necessary context for the current intersection of social media and medicine. The use of digital media in medicine is non-unique, as Dr. Gerald Weissman discusses in his book about medicine in the age of digitization. “Digital medicine” began in the early 2000s, immediately following the widespread popularization of cable television and the Internet [5]. For example, shows on television, such as The Dr. Oz Show and Sanjay Gupta’s various programs on CNN, gained large followings.

Dr. Mehmet Oz, a cardiothoracic surgeon at Columbia University, gained an enormous following by touting a new age of accessible medicine and advocating for alternative medicine, despite dubious scientific backing [6]. Oz shattered the predominant public perception of the time of a distant, intimidating doctor. Instead, the most famous doctor in America had personality and a show which closely resembled Oprah’s. However, Oz often sacrificed rigorous medical support, with only 46% of his claims in a sample of 400 shows supported by medical fact and over 15% contradicting mainstream medical beliefs, according to a study of Oz’s shows by Dr. Christina Korownyk at the University of Alberta [6]. However, the credibility and rapport he developed with his audience caused his “miracle pills” and other dubious advice to gain a following.

Of course, many asked why a reputable doctor who clearly understood appropriate medical practice would resort to recommending such treatments. The answer lies in digital media—for a television audience, “miracle weight loss pills” earned far more ratings than recommendations to eat healthier and exercise regularly [6,7]. While other doctors, like Sanjay Gupta, a neurosurgeon and CNN’s Chief Medical Correspondent, strove to maintain medical accuracy on television, the lack of sensationalized solutions and highly technical jargon limited popularity and viewership [6]. Even WebMD, the highest-trafficked website for healthcare online, has received much criticism for often providing more detailed information supporting drugs made by pharmaceutical companies who sponsor the website [5].

The superficiality of digital media affects the information conveyed on the media itself. For instance, in order to be a successful and profitable television show, Dr. Oz compromised medical accuracy. Thus, misinformation plagues digital medicine, as solutions to health problems are not always simple and facile [6,8]. Superficial explanations and recommendations can lead to unrealistic expectations placed on physicians and strain the doctor–patient relationship [1]. This inability to maintain both correct medical information and sustained viewership is a systematic problem in using traditional digital media platforms for medicine [8].

Despite high rates of misinformation, the increased accessibility of medicine on digital media has the potential to improve patient care and change the perception of medicine as cryptic or esoteric. This accessibility is extremely desirable in medicine, as highly technical terminology can alienate patients and detract from their care [1,9]. Thus, digital medicine in the age of television and early Internet introduced the dilemma of balancing accuracy and popularity, but made medicine much more accessible and could reduce the power imbalance that often exists between the healthcare provider and patient.

## 3. The Benefits of Twitter in Medicine

Despite clear flaws, many researchers extol digital media as a promising tool to involve patients more in their care and remove barriers to understanding medical treatment [1]. While television allows for many viewers to tune in to a medical professional on a show, the viewer involvement is passive, and only a few sources of information exist [5]. Social media, and particularly Twitter, has fundamentally different functionalities than television by greatly increasing the number of people who can contribute to discourse; anyone with a Twitter account can post public messages of 280 characters or less. Rather than information coming from a limited number of sources which must maintain viewership to stay on air, Twitter allows any medical expert to share their expertise by making an account [10]. Many accounts can provide medical information, while there can only be a certain number of medical shows on television. A recent study verified over 2000 doctors on Twitter based on their National Provider Identifier. These doctors all tweet more than once per day and have at least 300 followers each [10].

The first reason why Twitter has increased in popularity in medicine is that Twitter allows medical professionals to reach a broad audience ranging from other physicians to trainees to patients [4,7]. Anyone with a Twitter account can access the information. Moreover, on Twitter, the consumers of the information are no longer passive, as they can directly reply to tweets or endorse them with “favorites” or “retweets” [10]. Users can now use replies, retweets, and favorites to show which tweets that they view as useful and accurate. These reactions both tell medical professionals what content generates the most reaction and other users what content might be trustworthy. Even more, for each tweet, “hashtags” denote a topic around which users focus their discussions, which promotes communities and groups of tweets about the same disease or question types [2].

**Figure 1:**
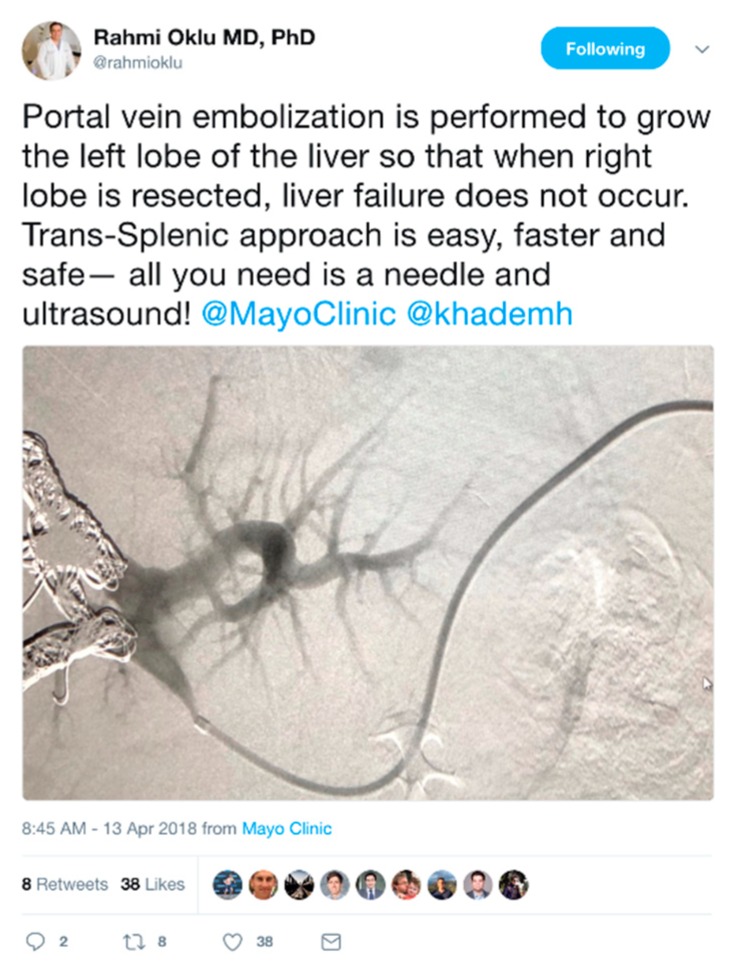
An example tweet from Dr. Rahmi Oklu, an interventional radiologist, on Twitter, in which he shares an interesting case study with patient data correctly anonymized.

Many medical professionals use Twitter for its potential to share and advance biomedical research. By allowing fellow researchers and physicians to connect and share research about new treatments, clinical problems that require further study, or interesting case studies, as seen in Figure 1.

Reducing barriers to collaboration in medicine is extremely valuable, as frequent constructive collaboration (i.e., more often and accessible than annual meetings) can greatly improve treatments and may range across disciplines into fields such as computer science and bioengineering [11]. For research articles, Twitter can also serve as a vetting arena for publications as tweets frequently provide critical commentary and opinions about newly published research. Tweeting about an article is much more feasible for researchers than commenting on the article website or crafting commentaries for publication.

Not only can Twitter connect biomedical researchers, it also serves to make research advances more accessible for physicians. Connecting researchers and clinicians is extremely important and useful, as clinicians can use new information they discover from this closer contact with researchers to guide decision-making about patient treatments in an ever-evolving field. Moreover, this exchange of clinical problems and new research can spur research in impactful and unique fields more openly and quickly than other strategies for collaboration [7]. Thus, the fundamental changes that Twitter provides are the two-way communication between the source and the consumer of information and a wider variety of information sources. Additionally, the two-way conversation on Twitter gives a voice to stakeholders who would not normally have one in traditional healthcare interactions.

**Figure 2:**
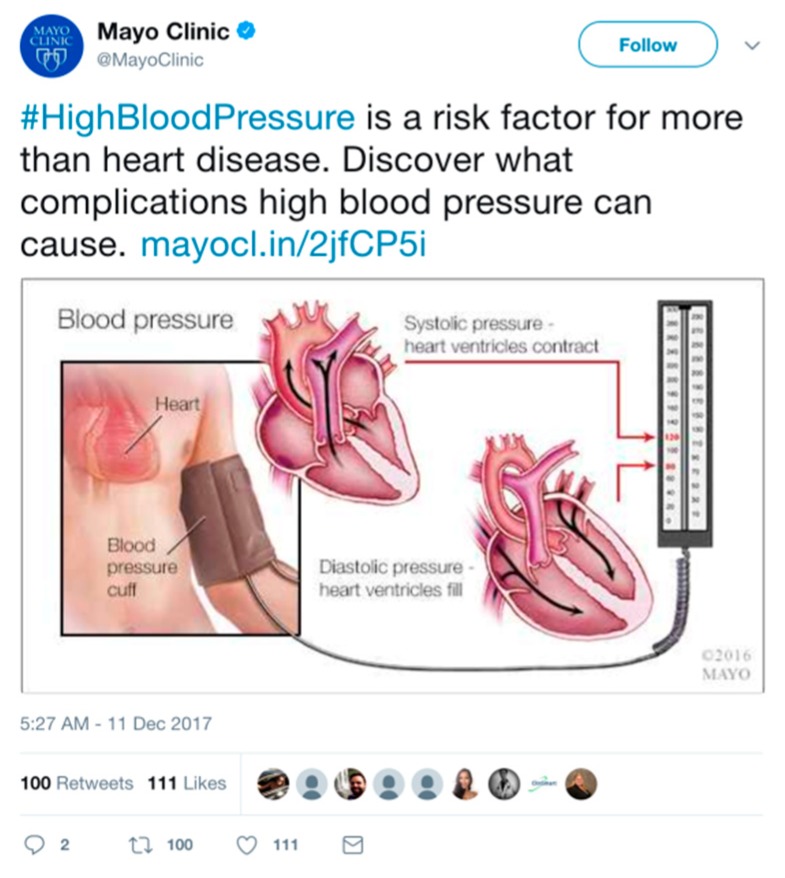
The verified Twitter account of Mayo Clinic shares information about #HighBloodPressure to inform followers about the condition’s health risks.

Through its ability to connect millions of people with public tweets, Twitter has the potential to revolutionize public health efforts, including disseminating health updates, sharing information about diseases, or coordinating relief efforts [9]. For example, tweets greatly helped aid workers in identifying locations to focus relief efforts in 2013 during Typhoon Haiyan. Also, Mayo Clinic posts regular updates on various medical conditions to educate its nearly 2 million followers, as seen in Figure 2.

A powerful example of the use of Twitter is that many of its benefits outlined above can potentially occur under a single post on the platform. For example, someone at a cardiology conference may respond to the tweet from Mayo Clinic about high blood pressure and discuss research on a new genetic correlation between high blood pressure and a specific gene. Another might respond by questioning the strength of the clinical outcomes of this study and other trials like it, prompting the sharing of papers. A practicing physician could respond with their experiences treating patients with hypertension. Another researcher might reply with a link to a healthcare conference where state-of-the art research on blood pressure management was presented. Moreover, a patient might ask these experts about the practicalities of dealing with a family member with hypertension. The diversity of expertise and backgrounds that can communicate on Twitter is unique, and this exchange of information can be extremely beneficial [12].

Ultimately, the sharing of information on Twitter has the potential to create a communicative and collaborative atmosphere for patients, physicians, and researchers. In this way, physicians practicing daily are connected to the newest research, researchers can identify clinical problems to solve, and patients can understand their conditions better [4,12,13]. This accessibility to information can greatly improve the quality of care delivered to patients through greater transparency and communication enabled by social media.

## 4. The Potential Risks of Using Twitter in Healthcare

While more accessible information on Twitter undoubtedly has the potential to benefit patient care, a major assumption made in this scenario is that all the information is accurate. Dr. Katherine Chretien and her research team at George Washington University studied the prevalence of misinformation in tweets about healthcare, and they found that about 20% contained inaccurate information [4]. The high rate of misinformation in these tweets hinders productive and helpful discussion on Twitter.

Moreover, no systematic process exists to check for misinformation on Twitter; instead, it relies on “crowdsourcing,” or voluntary contributions from medical professionals who try their best to correct inaccurate statements [4]. Thus, it is fairly easy for “non-experts, the incorrectly informed, or commercial interests” to present their opinions as fact and appear to have the same credibility as unbiased medical professionals [7]. Such scenarios can be particularly problematic for patients or students, who may have difficulty distinguishing credible from non-credible sources [7].

**Figure 3:**
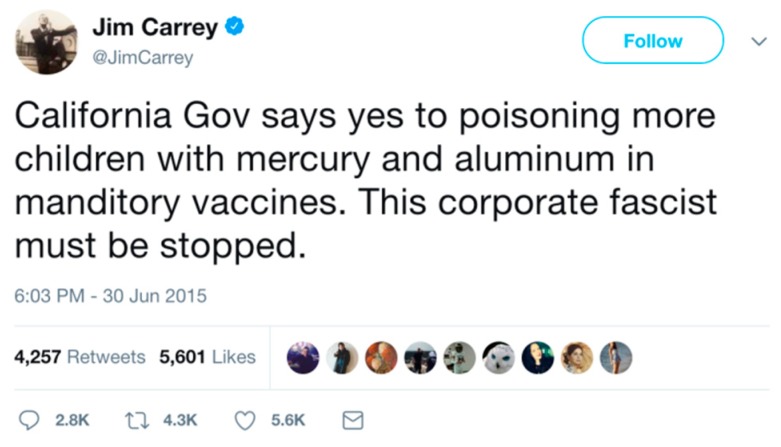
Tweet from Jim Carrey’s verified account lacking credible medical evidence on vaccines.

One example of dangerous misinformation caused by non-experts sharing opinions without scientific backing is the celebrity anti-vaccination movement. Several celebrities, most notably Jenny McCarthy and Jim Carrey, have tweeted about how vaccines have been linked to autism, despite no medical basis in fact, as seen in Figure 3.

As a celebrity comedian, Jim Carrey has a verified account, but has no formal expertise on the subject of vaccines. Twitter can be an “echo chamber of ideas,” representing shared opinion rather than balanced facts, due to the ease of quoting or retweeting messages [5]. The anti-vaccine movement has gained traction, in part because of misguided tweets and the echo effect. As a consequence, some areas of the United States are experiencing resurgences in previously-eradicated diseases such as measles [5].

Celebrities rebuking vaccinations are egregious examples of non-experts commenting on medical phenomena, but the general issue of distinguishing representatives of commercial interests from unbiased physicians is as difficult and problematic [7]. The matter is made worse with anonymous and unmarked accounts, whose claims and expertise are not easily verifiable [14]. Thus, a potential risk is the difficulty in identifying doctors on Twitter definitively and trusting certain accounts. In addition, due to the small number of characters required, tweets are often brief and must omit key information, which can sometimes create miscommunications. Particularly for complex research, it is difficult to discuss nuances, caveats, and limitations in tweets [11]. For researchers, this problem can manifest in patients misunderstanding new advances. For doctors, these risks can be extremely dangerous, as statements from a verified doctor may be taken as medical advice [15].

Another issue with sharing information on Twitter for medicine is that Twitter can transmit such a high volume of information. The amount of information makes it nearly impossible to absorb and filter all relevant tweets [7]. The same qualities that generate discussion may also bring an overwhelming amount of information. For nearly every hashtag, many professionals and non-experts may provide their insight, with illegitimate and inaccurate claims reducing the impact of the accurate expert information [7,16]. Moreover, when the accuracy of information is challenged, replies and comments may be overlooked, so the final conclusions and resolutions may go unnoticed. In these situations, more information is not necessarily better.

Moreover, lapses in professionalism are also serious potential problems with Twitter, as with other forms of social media. Previous studies have found high rates of unprofessional tweeting from accounts clearly identified as doctors [4,15]. Unprofessional behavior included profanity, complaints about patients, violations of patient privacy, and conflicts of interest. These lapses are due to Twitter’s nature and users’ inexperience.

The final potential harm of using Twitter in healthcare is the use of physician time. While making physicians and their knowledge more accessible on social media is valuable, these activities also take time out of the day for doctors. Moreover, over 80% of tweets from verified doctors are published during the workday, from 9 a.m. to 5 p.m.; this time on Twitter takes away from other potentially more important activities for daily patient care [2]. Physician resistance has been demonstrated explicitly, through commentaries about the lack of “return on investment” of involvement in social media due to lack of time and fear of distraction from their “core academic or professional activities” [16]. If a low percentage of doctors opt into social media healthcare discourse, then advice and accurate information on Twitter will be rare.

For the sharing of general information on Twitter, the risks involved with using Twitter for healthcare discourse include high rates of misinformation, difficulties verifying the credibility of sources, overwhelmingly high volumes of information available on Twitter, concerns about professionalism, and the opportunity cost of using physician time. In order to compare the risks and benefits of the use of Twitter in medicine, this paper examines the current uses of Twitter in medicine to propose a solution to maximize benefits and minimize risks.

## 5. Current Uses of Twitter in Healthcare

**Figure 4:**
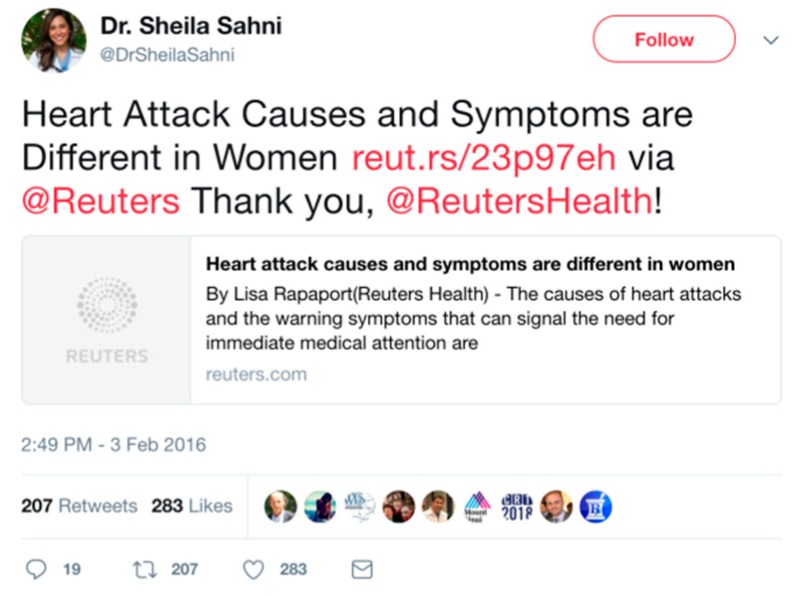
An example tweet from Dr. Sheila Sahni, a physician and experienced Twitter user.

With over 2000 healthcare providers on Twitter who tweet more than once per day and have at least 300 followers, Twitter is becoming a major part of modern medicine because of the benefits it can provide. Many tweets from medical accounts share general information for public health or new research about treatments or technology. For instance, in Figure 4, Dr. Sheila Sahni, an interventional cardiologist with approximately 7000 followers and 8500 tweets, shares a recent study about the different causes and symptoms of heart attacks in women compared to men. As discussed earlier, this type of tweet maximizes the benefits of Twitter by enabling the sharing of new research and informing patients, without risking any patient privacy.

However, less-experienced physicians on Twitter have tweeted comments about interactions with patients, pictures of X-rays, and cropped images of notes from real patients. While some case studies can be instructive to a general audience, extreme precaution must be taken to remove identifying patient data from the tweets to prevent privacy violations. One study reported that out of a group of 500 doctors who had over 200 followers, more than 5% of tweets were violations of patient privacy [4]. These breaches of patient privacy are unprofessional and illegal.

Moreover, Twitter accounts for doctors with fewer than 300 followers rarely had the disclaimer “Tweets are my opinions not medical advice”, which is important to inform the general public about the content of the tweets and prevent doctors from liabilities [15]. Thus, investigating tweets from health professionals revealed that doctors who are more experienced and more proficient in the use of Twitter tend to have more followers. These doctors also tend to use Twitter to maximize the benefits described earlier, such as making public health information accessible, and to minimize privacy, misinformation, and unprofessionalism. 

Twitter can be an excellent tool for sharing research and connecting physicians to experts in other fields. For instance, research conferences have extensively leveraged Twitter’s capabilities to generate interest and maximize impact [12]. Physicians can also learn of clinical trials through Twitter and enroll patients which would never have had access to care otherwise [2]. Additionally, Twitter can impact patient quality of care and accessibility to health information.

Other clear applications of Twitter to medical research is the application of a multi-tiered approach of sentiment analysis, data-mining, and artificial intelligence to analyze tweets from the general population [17,18]. This real-time information can inform resource allocation for researchers and treatment plans for physicians [3]. Previous studies have shown the predictive power of Twitter for public health ranging from tracking infectious disease outbreaks, natural disasters, drug use, and more [3,4,14,15,16,17,18,19,20]. While computational power was previously a bottleneck for mining massive datasets, current advances in graphics processing units (GPUs) have enabled efficient instantaneous mining and analysis.

Examining the current uses of Twitter in medicine has demonstrated that many doctors are using Twitter effectively to share information about research among their colleagues and collaborators and information about public health for the public. However, some doctors know much less about the appropriate uses of Twitter. This knowledge gap is problematic and suggests little formal education on social media practices amongst physicians.

## 6. Proposed Solutions

Medical professionals must constantly communicate with patients, peers, and researchers. As social media is becoming an integral part of communication, medical professionals of the twenty-first century must acquire new skills on social media. Concerns with misinformation, privacy, and unprofessionalism necessitate clear guidelines for physicians on Twitter.

Despite this need for clear guidelines, the current guidelines from the American Medical Association (AMA) are vague, telling doctors to “use their common sense” [4,21]. The high rates of unprofessionalism and misinformation, along with the lack of consistent identifiers for healthcare professionals in the “bios” of their Twitter accounts, show a lack of formal social media education among physicians [15]. This lack of knowledge is not surprising, as traditional training for healthcare professionals does not include effectively leveraging social media for clinical or research purposes.

Thus, medical professionals must be formally trained on appropriate and effective social media use. This training is a necessity as social media becomes a bigger part of our daily lives and medicine. Similar initiatives have informed collegiate and professional athletes, who have now become major figures on social media [22]; formal instruction integrated in medical school and throughout training could even be more successful, as all medical professionals go through similar curricula.

Moreover, the AMA should enact new guidelines for the use of social media by medical professionals. These guidelines should emphasize that health professionals should maintain separate personal and professional accounts, avoid profanity, racist or sexist statements, or sexual references, respect the privacy and anonymity of patients, avoid promoting specific products or medication, and include clear statements of affiliations and conflicts of interest in Twitter account profiles [7,16]. Health professionals should try to combat misinformation as vigorously as they propagate information. Additionally, the guidelines should tell doctors to re-read their tweets before posting, as reflecting on the tweet before posting is crucial to protect patient privacy and ensure professional behavior. Moreover, using the Twitter “bio” effectively and professionally is crucial to allow patients to find credible sources of information.

While the proposed solution to educate medical professionals on social media etiquette during their training has potential to mitigate risks associated with physicians on social media, educating doctors should be an iterative and continuous process. Implementing any education program, which is better than the current complete lack of education, is a step in the right direction, but only a start. Moreover, the distinction between types of healthcare professionals, including doctors, physician assistants, nurses, and others, has not yet been addressed in the literature and requires further study for a more nuanced solution.

Ultimately, Dr. Oz, WebMD, and Twitter all share the common goal of changing the perception of medicine from a black box to something more accessible—to allow patients to understand their conditions and make informed decisions. The use of Twitter in healthcare has the potential to allow patients to be more informed about their own health, but must balance accessibility with misinformation.

## References

[1] Cayton H. (2006). The alienating language of health care. J. R. Soc. Med..

[2] Brady R.R.W., Chapman S.J., Atallah S., Chand M., Mayol J., Lacy A.M., Wexner S.D. (2017). #colorectalsurgery: #colorectalsurgery. Br. J. Surg..

[3] Raghupathi W., Raghupathi V. (2014). Big data analytics in healthcare: Promise and potential. Health Inf. Sci. Syst..

[4] Chretien K., Azar J. (2011). Terry Kind Physicians on twitter. JAMA.

[5] Weissmann G. (2012). Epigenetics in the Age of Twitter: Pop Culture and Modern Science.

[6] Korownyk C., Kolber M.R., McCormack J., Lam V., Overbo K., Cotton C., Finley C., Turgeon R.D., Garrison S., Lindblad A.J. (2014). Televised medical talk shows—What they recommend and the evidence to support their recommendations: A prospective observational study. BMJ.

[7] Choo E.K., Ranney M.L., Chan T.M., Trueger N.S., Walsh A.E., Tegtmeyer K., McNamara S.O., Choi R.Y., Carroll C.L. (2015). Twitter as a tool for communication and knowledge exchange in academic medicine: A guide for skeptics and novices. Med. Teach..

[8] Jotterand F. (2005). The Hippocratic Oath and Contemporary Medicine: Dialectic between Past Ideals and Present Reality?. J. Med. Philos..

[9] Wiederhold B.K. (2012). ICT: This Transformer Isn’t Science Fiction. Cyberpsychol. Behav. Soc. Netw..

[10] Meskó B. (2013). Conclusions. Social Media in Clinical Practice.

[11] Widmer R.J., Engler N.B., Geske J.B., Klarich K.W., Timimi F.K. (2016). An Academic Healthcare Twitter Account: The Mayo Clinic Experience. Cyberpsychol. Behav. Soc. Netw..

[12] Thompson M.A., Majhail N.S., Wood W.A., Perales M.-A., Chaboissier M. (2015). Social Media and the Practicing Hematologist: Twitter 101 for the Busy Healthcare Provider. Curr. Hematol. Malig. Rep..

[13] Pemmaraju N., Thompson M.A., Qazilbash M. (2017). Disease-specific hashtags and the creation of Twitter medical communities in hematology and oncology. Semin. Hematol..

[14] DeCamp M., Koenig T.W., Chisolm M.S. (2013). Social Media and Physicians’ Online Identity Crisis. JAMA.

[15] Gagnon K., Sabus C. (2015). Professionalism in a Digital Age: Opportunities and Considerations for Using Social Media in Health Care. Phys. Ther..

[16] Melvin L., Chan T. (2014). Using Twitter in Clinical Education and Practice. J. Grad. Med. Educ..

[17] Baumgartner P., Peiper N. (2017). Utilizing Big Data and Twitter to Discover Emergent Online Communities of Cannabis Users. Subst. Abuse Res. Treat..

[18] Kuehn B.M. (2015). Twitter Streams Fuel Big Data Approaches to Health Forecasting. JAMA.

[19] Lee K., Agrawal A., Choudhary A. (2013). Real-Time Disease Surveillance Using Twitter Data: Demonstration on Flu and Cancer.

[20] Sakaki T., Okazaki M., Matsuo Y. (2010). Earthquake Shakes Twitter Users: Real-Time Event Detection by Social Sensors.

[21] Kind T. (2015). Professional Guidelines for Social Media Use: A Starting Point. AMA J. Ethic.

[22] Ballouli K., Hutchinson M. (2010). Digital-Branding and Social-Media Strategies for Professional Athletes, Sports Teams, and Leagues: An Interview with Digital Royalty’s Amy Martin. Int. J. Sport Commun..

